# Indoor Positioning System Using Dynamic Model Estimation

**DOI:** 10.3390/s20247003

**Published:** 2020-12-08

**Authors:** Yuri Assayag, Horácio Oliveira, Eduardo Souto, Raimundo Barreto, Richard Pazzi

**Affiliations:** 1 Institute of Computing, Federal University of Amazonas, Manaus 69067-005, Amazonas, Brazil; horacio@icomp.ufam.edu.br (H.O.); esouto@icomp.ufam.edu.br (E.S.); rbarreto@icomp.ufam.edu.br (R.B.); 2 Faculty of Business and Information Technology, Ontario Tech University (UOIT), Oshawa, ON L1H 7K4, Canada; richard.pazzi@uoit.ca

**Keywords:** indoor positioning systems, bluetooth low energy, path-loss model, localization systems

## Abstract

Indoor Positioning Systems (IPSs) are used to locate mobile devices in indoor environments. Model-based IPSs have the advantage of not having an exhausting training and signal characterization of the environment, as required by the fingerprint technique. However, most model-based IPSs are done using fixed model parameters, treating the whole scenario as having a uniform signal propagation. This might work for most small scale experiments, but not for larger scenarios. In this paper, we propose PoDME (Positioning using Dynamic Model Estimation), a model-based IPS that uses dynamic parameters that are estimated based on the location the signal was sent. More specifically, we use the set of anchor nodes that received the signal sent by the mobile node and their signal strengths, to estimate the best local values for the log-distance model parameters. Also, since our solution depends highly on the selected anchor nodes to use on the position computation, we propose a novel method for choosing the three best anchor nodes. Our method is based on several data analysis executed on a large-scale, Bluetooth-based, real-world experiment and it chooses not only the nearest anchor but also the ones that benefit our least-square-based position computation. Our solution achieves a position estimation error of 3 m, which is 17% better than a fixed-parameters model from the literature.

## 1. Introduction

Today´s most commonly used positioning system is the Global Navigation Satellite Systems (GNSS), which includes the Global Positioning System (GPS). They allow people to navigate from place to place through applications such as Google Maps, Waze, and Apple Maps. However, the satellite signals are easily blocked by buildings, decreasing its accuracy, and making its usage limited to outdoor environments [1]. For this reason, Indoor Positioning Systems (IPSs) have been proposed to allow the location of mobile devices indoors, using the local infrastructure. IPSs have been drawing the attention of many companies since it allows the development of several interesting applications, such as monitoring the position of the elderly in retirement homes, monitoring children in schools, assisting customers in supermarkets, and tracking patients and equipment in hospitals [2].

Many IPSs have been proposed in the literature, but to date, no system has been established as standard since each one has its pros and cons. Positions can be estimated using several data sources, such as the Angle of Arrival (AoA), Time of Arrival (ToA), Time Difference of Arrival (TDoA), and the Received Signal Strength Indicator (RSSI). However, most IPS solutions are based on the RSSI due to its low cost and high availability since they can use signals from WiFi and Bluetooth, both of which are available in most mobile devices [3].

The RSSI can be used to estimate the distance between two devices since there is a decrease in the signal strength as the distance increases [4]. However, the RSSI is sensitive to environmental noises, such as obstacles from furniture and walls, people’s movements, and opening or closing doors, all of which can cause a high signal variation making it difficult to convert the signal strength to distance accurately [3]. Consequently, the position estimation of the mobile device is affected. Many wireless communication technologies can be used in IPS, such as WiFi and Bluetooth, as mentioned, but also Radio Frequency Identification (RFID), Ultra-Wide Band (UWB), ZigBee, and others. WiFi has greater prominence since it is the most used technology and, therefore, more easily found indoors, which results in no need for extra hardware [5]. However, WiFi consumes more energy, making it unfeasible for small, low-power devices [6]. Thus, with the development of Bluetooth Low Energy (BLE), it has become increasingly common to employ this technology due to its low energy consumption, ease of deployment, and low cost [7].

IPSs can be classified into two categories: fingerprint-based and model-based. Fingerprint-based IPSs use an extensive and exhaustive training of reference points in the scenario to feed a machine learning algorithm that will later be used to localize the mobile devices. The created signal map is susceptible to changes in the environment [8] and is unfeasible to be generated and maintained for larger scenarios. On the other hand, model-based IPSs require only some information from the scenario, such as the coordinates of the anchors (reference nodes) and, in some cases, a simple collection of signal data, to create a better signal propagation model for the scenario. The model-based IPSs have two main phases: model data gathering and position computation [9]. In the first phase, RSSI samples are taken to obtain the signal propagation model (also known as path loss model) that characterizes the signal strength in the scenario as the distance increases. This is the most sensitive step since it depends directly on the measured RSSI values in the real environment, which are known to have a high variance, and they will also affect the estimated distances accuracies [4]. In the position computation phase, the positions of the mobile devices are computed using the distances estimations and the known position of the anchors. This computation is usually done by some optimization algorithms, such as the least-squares [10].

Most model-based IPSs proposed in the literature [10,11,12,13] use the same fixed model parameters for the whole scenario. This fixed model considers that the signals behave uniformly over the whole scenario. However, this is not the case, especially for medium to large-scale scenarios in which the signal behavior changes from place to place depending on the obstacles and other environment variables. In this paper, we propose PoDME (Positioning using Dynamic Model Estimation), an IPS that uses a signal propagation model with dynamically estimated parameters to improve the distance computation between the mobile device and the anchor nodes. Our main idea is that these dynamically estimated path loss parameters correspond more closely to the region’s characteristics the packet we want to localize was sent from, improving the accuracy of the estimated distances. Also, since our solution depends highly on the selected anchors used on the position computation, we propose a novel method for choosing the three best anchors that focus not only on the nearest anchors but also the ones that benefit the least-squares-based position computation.

The main goal of our proposed IPS is to provide location information for small, battery-powered devices to be worn by people inside buildings, such as the elderly in retirement homes. These mobile devices are required to be operated by a single, small battery while also having other sensors. Thus, in our experiments, we decided to use the Bluetooth Low Energy (BLE) technology. We implemented our solution in a real-world, large-scale testbed and compared its performance to different variations of a fixed model-based IPS, as used by most model-based solutions in the literature. Our results show an average error of 3 m, a 17% improvement compared to the best experimented parameter of a fixed model-based IPS, which had an error of 3.6 m.

The remainder of the paper is structured as follows: Section 2 presents our related work. In Section 3, we present our proposed PoDME solution. In Section 4, we show the performance evaluation of our solution in a real-world testbed. In Section 5, we briefly discuss some applicability issues of our solution. Finally, in Section 6, we present our conclusions and future work.

## 2. Related Work

Current proposed IPSs are mainly based on Bluetooth [14,15,16,17] or WiFi [5,13,18,19] due to the already available infrastructure and already supported devices, which reduces the implementation cost. With the advances in Bluetooth Low Energy (BLE), an improved version of Bluetooth, it has been possible to obtain a more significant reduction in cost while also reducing the power consumption, ensuring an increased lifespan for the devices [16]. Also, BLE introduced the advertisement packets, which are data packets used mainly for positioning, in which the receiving devices are required to report the Received Signal Strength Indicator (RSSI).

Regarding the technique used to estimate the mobile nodes’ position, current IPSs can be classified into fingerprint-based and model-based. In the fingerprint technique [20,21,22,23], training of the whole scenario is required to generate an RSSI database that will be used by machine learning algorithms to estimate positions in the online phase. The positioning system developed by [20], called RADAR, is a classic work in the area because it was one of the first to create a signal map using RSSI. The solution proposed by the authors resulted in an accuracy of 2 m to 3 m. The fingerprint method used in RADAR is more accurate than model-based solutions, but it is time-consuming since it requires the characterizations of the environment through the signal map. Also, this method is susceptible to changes in the environment, requiring a new characterization in this case.

Among the RSSI-based methods, the nearest-anchor method is possibly the most simple solution. It considers that the node’s position is the same as the anchor with the highest signal strength [17], since the higher the signal strength, the closer the mobile node. This method’s accuracy is directly linked to the organization of anchor nodes in the scenario, requiring a large anchors number for larger environments [24]. The works of [9,17] compare the nearest-anchor with other model-based techniques.

As with the fingerprint-based IPSs, the main information used in model-based IPSs is the RSSI. However, it is well-known that the signal strength value varies widely, either due to the nature of the wireless channel or due to obstacles in the environment [12,25]. problem is worse in the packet losses presences, especially when using BLE advertising packets, in which only half of the packets are usually received. To decrease the signal variation, some authors suggest the use of filters such as the mean filter [26], particle filter [27], and Kalman filter [28]. The sliding window filter also proved to be a simple solution with some of the best results [26].

Model-based IPSs use propagation models to transform the RSSI value in the distance between the sender and receiver. Such propagation models try to model the behavior of the signal in relation to the variation of distance. In [14], the authors developed an empirical propagation model using the RSSIs collected from a real-world experiment to estimate the distance based on the signal strength. However, the proposed propagation model is valid only for the experimented site.

Li et al. [4] proposed an IPS that uses a distance-based RSSI adjustment model to correct signal losses in the environment. The work compares three main propagation models: log-distance, back propagation neural network (BPNN), and back propagation neural network with a particle swarm optimization (PSO-BPNN). Their experiment is performed in a small-scale scenario with an area of 9 × 6 m², consisting of four anchor nodes, an Android-based mobile device, and a gateway. The proposed solution resulted in a root mean square error (RMSE) of 2 m when using PSO-BPNN. However, these results were obtained by evaluating only 8 reference points and without considering the corners of the scenario as well as other regions with more complex signal behaviors.

After using the propagation model to convert RSSI to distance, the position computation is done using algorithms such as maximum likelihood and least-squares [1,11,13,29], which estimate the location of the target by representing the distance as the radius of the circles formed by the anchors or by representing triangles. The system proposed by [30], called AcMu, explores the static behavior of mobile devices, using the regression of the partial least-squares to update the signal map with data from the user’s mobile devices. The system uses the signal intensities’ readings in real-time received at the reference points to update the model.

In [31], it is proposed a positioning system that uses signal information among anchor nodes to obtain the exponent of the path loss model for the environment. This proposal uses the average distance among all anchors to estimate the location. Similarly, the work of [32] uses the relative values between pairs of anchors and the site map information, such as walls and obstacles, to decrease the impact of the building infrastructure on the position computation. They evaluate the number of anchors impact on the system performance and the effect of techniques to decrease the signal variation. Their results show an average error of 2.8 m.

An IPS based on signal diversity and least squares is proposed in [13]. In the proposed solution, the RSSI noise is first filtered using an adaptive Kalman filter to decrease the variability. Next, the values of two functions are computed using a channel filter to obtain the degree of correspondence between the RSSI values on the different channels to prevent the distance estimation between nodes from falling into local optimum, which would prevent them from reaching the global optimum. The experiments were performed in a small-scale area of 10 × 10 m² with three Bluetooth-based anchors. Their results show an average error of 1.5 m.

Finally, in [15], the authors proposed a Bluetooth-based IPSs aimed at improving accuracy while reducing both energy consumption and total cost. The system does not perform signal characterization for the different regions and uses a fixed path loss exponent value. The results show an error of 4.6 m in 90% of the time for a scenario with an area of 16.50 × 17.60 m². For this, their proposal focused on frequency diversity, signal filtering using the Kalman filter, and a weighted least squares method (WLS), without considering the form of anchors organization. WLS works by increasing the weights of receivers that are closer to the emitter. Their work compares three different propagation models: the International Telecommunication Union (ITU) model, the log-distance model with shadowing, and a fitted empirical model.

Our proposed PaDME solution differs from all of the mentioned solutions. First, our solution is implemented in large-scale scenarios compared to the scenarios from the previous works. Second, our model-based IPS uses dynamic parameters that allow selecting the best values of the path loss exponent that characterize the region of the mobile node we want to localize. This results in better accuracies for the estimated distances, unlike the fixed parameters for all regions of the environment used by the mentioned solutions. Third, we propose a novel method for choosing the best anchor nodes that benefit the least-squares-based position computation by using both the highest RSSI values and the similarity to equilateral triangles, which increases considerably the positioning accuracy. The details of our proposed solution are described in the next section.

## 3. PoDME—Positioning Using Dynamic Model Estimation

**Figure 1:**
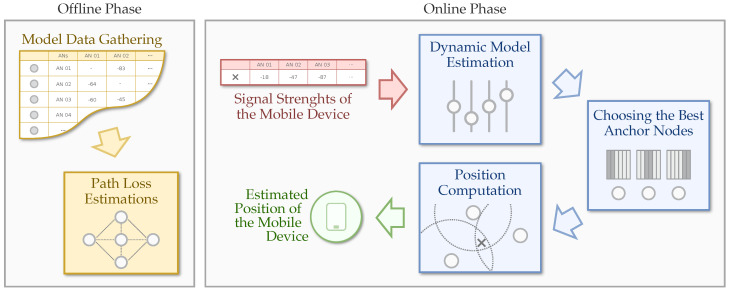
Phases and components of our PoDME architecture.

In this section, we present our proposed PoDME architecture. Figure 1 shows the components of our system. In the offline phase, we performed model data gathering and obtained the path loss estimates between the anchor nodes. In the online phase, we perform the position computation using the RSSI values, choosing the best anchor nodes, and using the path loss estimates obtained in the offline phase to improve the distance mapping between the mobile device and the anchor nodes. All of these components are detailed in the next sections.

### 3.1. Model Data Gathering

Our solution’s first step is to collect some RSSI samples to obtain signal behavior in the environment. Most proposed model-based solutions in the literature require some signal information to fit their models. In some solutions, it is done automatically [31,32], in others, manually [3,4].

Our goal is to create a database containing the RSSI values that represent the distances among anchor nodes and use it in the next sections to estimate the path-loss in different regions of the environment. We consider that we know the positions of the anchors, which is common in these type of IPS [9,12,13,33] and, thus, we can quickly obtain the distance among anchors by computing the Euclidean distance, as shown in Equation (1).

(1) $$d_{ab}=\sqrt{{\left(x_{a}-x_{b}\right)}^{2}+{\left(y_{a}-y_{b}\right)}^{2}}$$

where $d_{ab}$ is the distance between Anchor${}_{a}$ and Anchor${}_{b}$.

Now we need to know the RSSI behavior among the anchors. Therefore, in this part of our PoDME solution, we propose a simple data gathering methodology in which a person gets one or more mobile devices and physically positions himself below or near an anchor node. From that location, the mobile devices start sending packets. These packets will be received by all nearby anchors with different RSSI values, depending on their positions and the characteristics of the environment in that region. This step is repeated for all anchors in the scenario.

Since we are using Bluetooth advertising packets, we need to filter the RSSI values before using them. Besides the known RSSI variation, the use of Bluetooth advertising packets has another problem: packet loss. It happens because the nodes send these packets in three different channels and, thus, the receiver needs to alternate among these channels constantly. In our experiments, it is common for the anchors to lose nearly 50% of the packets. To solve this problem, we used the highest RSSI value from the last 10 seconds to smooth out RSSI on each measurement.

**Figure 2:**
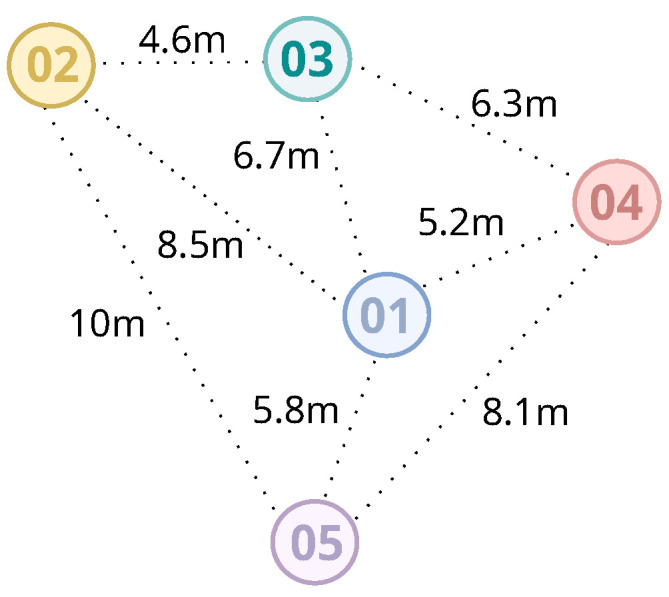
Real distances among anchor nodes.

As a result, after taking the measurements, we obtain the RSSI values of all anchors to all of their neighbors. However, since we need only one RSSI value, we take several RSSI samples and use the average RSSI as the value that represents the signal among each anchor node. In Table 1, we have an example of a Model Data Gathering containing the RSSI values between 5 anchors. In the table, not filled cells mean that we have no signal between the two anchors, which indicates that they are far from each other. The relationship between RSSI and distance can be seen in Table 1 and Figure 2. For example, the Anchor${}_{1}$ is positioned at a distance of 5.2 m from Anchor${}_{4}$ and has an RSSI average of −85 dBm.

### 3.2. Path Loss Estimations

Now that we have the distances among anchor nodes, given by the map information and Equation (1), and also the RSSI values among these anchors, collected in the previous section, we can estimate the best parameters for a Path-Loss Model. Given that the distances and RSSIs among the anchors change, we will have a different Path Loss Model for each pair of anchors.

A Path Loss Model predicts the fading of a signal as it travels a given distance. Such behavior of the signal variation with respect to distance is usually modeled by a logarithmic equation. Several propagation models are proposed in the literature to better relate the signal, distances, and obstacles. However, the Logarithmic Distance Path Loss Model is the most known and used [4,12,34]. This model is given by the following equation:

(2) $$R\left(d\right)=R_{0}-10\eta \log\left[10\right]\frac{d}{d_{0}}+X_{\sigma }$$

where R(d) is the RSSI value measured at distance *d*, $R_{0}$ is the RSSI value measured at distance $d_{0}$, η is the path loss exponent, i.e., a signal loss rate related to the environment and, finally, $X_{\sigma }$ is a zero-mean Gaussian random variable [33] that models the RSSI variation. For the $d_{0}$ model parameter, a distance of 1 m is commonly used in the literature [35,36]. Thus, $R_{0}$ is the RSSI at 1 m, and we will use a fixed value based on the data gathered from the previous section. Also, even if this value is not the optimal one, this error is easily corrected by the least-squares technique, as explained in the last part of our solution.

Thus, the only environment-dependent variable of the model is the path loss exponent (η). From Equation (2), we can compute this parameter for each pair of anchors:

(3) $$\eta_{ab}=\frac{R_{0}-R_{ab}}{10\times \log\left[10\right]\frac{d_{ab}}{d_{0}}}$$

where $\eta_{ab}$ is the path loss exponent between Anchor${}_{a}$ and Anchor${}_{b}$, $R_{ab}$ is the RSSI between them, as shown in Table 1 and, finally, $d_{ab}$ is the distance between them, as in Equation (1). Please note that we ignored the parameter $X_{\sigma }$, since $R_{ab}$ is an averaged value from several samples.

**Figure 3:**
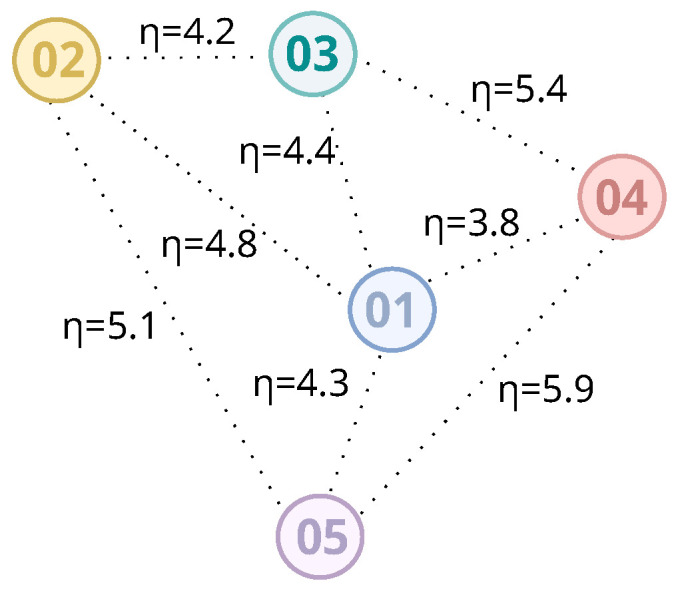
Path-loss exponents among anchor nodes.

Therefore, we can estimate all of the path loss exponents in the scenario based on the distance and RSSIs among anchors. A higher $\eta_{ab}$ would indicate a higher number of obstacles and other fading factors between Anchor${}_{a}$ and Anchor${}_{b}$, while two anchors with direct visibility from each other would result in a lower path loss exponent. Figure 3 and Table 2 show examples of path loss exponents for the values in Figure 2 and Table 1. It is worth noting how these values change from one anchor node to another. When we establish the path loss exponent between two anchor nodes, we are characterizing the signal behavior in the region in which they are located, based on their respective positions on the map. Thus, when a mobile device sends a packet and has the signal strengths collected, the distance estimation is made using the respective values of path loss exponents between the nearest anchors.

### 3.3. Choosing the Best Anchor Nodes

The next part of our proposed PoDME solution is executed every time we have a new sample to locate, i.e., a mobile node sent a packet that was received by several anchor nodes, and we need to estimate the mobile position. In this part, we will choose, among all anchors that received the packet, which ones we will use in the next parts of our solution.

The main reason for choosing the best anchors is because the Position Computation part of our solution, detailed in Section 3.5, uses the least-squares technique to find the position of the target. This technique uses distances computed using our Dynamic Model Estimation (detailed in the next section) to find the most consistent position. However, using all of the anchors information, leads to greater errors, since we will use information from faraway anchors, that will have higher distance errors. On the other hand, if we use only the information from the three closest anchors (based on their RSSI values), their positions may be somewhat collinear, which greatly decreases the estimated position accuracy. Thus, in this part, we aim at choosing the closest three anchors that are far from being collinear.

For this, the first step is to sort the list of anchors that received the mobile node packet by their RSSI values in such a way that the closest anchors will be at the beginning of the list. Then, we get the three closest anchors and check their positions against an equilateral triangle similarity filter that will be detailed in the next paragraphs. This test will tell how close the anchors positions form an equilateral triangle since this would be the farthest from them being collinear and, thus, the best-case scenario. If the three closest anchors pass the filter, they will be the chosen anchors for the next parts of our architecture. However, if the anchors fail the check, i.e., they are somewhat collinear, we ignore them and test the second closest anchors, and so on. If we reach the end of all anchors combinations and they all have failed the similarity checker, we then fall back to the three nearest anchors.

Given the positions of three anchors (Anchor${}_{a}$, Anchor${}_{b}$, Anchor${}_{c}$), our equilateral triangle similarity checker starts by computing the internal angles of the triangle formed by the anchors positions:

(4) $$\alpha =\left(\frac{\cos{d_{ac}}^{2}+{d_{bc}}^{2}-{d_{ab}}^{2}}{2\times d_{ac}\times d_{bc}}\right)\times 180/\pi$$

(5) $$\beta =\left(\frac{\cos{d_{ab}}^{2}+{d_{bc}}^{2}-{d_{ac}}^{2}}{2\times d_{ab}\times d_{bc}}\right)\times 180/\pi$$

(6) $$\gamma =\left(\frac{\cos{d_{ab}}^{2}+{d_{ac}}^{2}-{d_{bc}}^{2}}{2\times d_{ab}\times d_{ac}}\right)\times 180/\pi$$

Then, we compute how far from 60° these angles are since an equilateral triangle has three internal angles of 60°:

(7) $$\Delta =\sqrt{{\left(\alpha -60\right)}^{2}+{\left(\beta -60\right)}^{2}+{\left(\gamma -60\right)}^{2}}$$

The closer ∆ is from zero, the closer the anchors are to form an equilateral triangle. The threshold chosen in our proposed solution has a value of 75. Thus, if the three anchors at the beginning of the list have a ∆ between zero and 75, then they can be used in the position calculation, otherwise, we evaluate other sets. This threshold was obtained empirically, as will be shown in our performance evaluation in Section 4.

### 3.4. Dynamic Model Estimation

As shown in Section 3.2, the only environment-dependent variable of the log-distance model is the path loss exponent (η). As mentioned, in most model-based IPSs [10,11,13,15] this parameter is fixed for the whole scenario. This approach is not recommended for large-scale scenarios since the path loss exponent changes from place to place depending on the obstacles and other environment variables. In our PoDME solution, we propose the use of a dynamically computed path loss exponent, in such a way that this value corresponds more closely to the characteristics of the region the packet we want to localize was sent from.

In the last section, we chose the best three anchors to be used to locate that specific packet sent by the mobile node. These anchors are closer to the mobile node and, thus, their information can be used to estimate the local path loss exponent to be used in the position computation. The goal is to use the average value of the path loss exponent among the neighbors anchors to represent the region where the mobile device is located. For this, we use the Path Loss Estimations, computed in Section 3.2. There, we computed the path loss exponent among all pairs of anchors. Since in the next section, we will need three distance estimations, one for each anchor, we will compute three different path loss exponents. Thus, the final path loss exponent, for a given anchor, will be the average exponent between this anchor and the other two:

(8) $$\begin{array}{cc} \eta_{ma} & =(\eta_{ab}+\eta_{ac})/2 \end{array}$$

(9) $$\begin{array}{cc} \eta_{mb} & =(\eta_{ba}+\eta_{bc})/2 \end{array}$$

(10) $$\begin{array}{cc} \eta_{mc} & =(\eta_{ca}+\eta_{cb})/2 \end{array}$$

where $\eta_{ma}$ is the path loss exponent that will be used to compute the distance between the mobile node and Anchor${}_{a}$, and so on.

As we can see, the model parameters estimated in this part of PoDME depend mainly on the anchors that heard the packet from the mobile node. Thus, this parameter can change for each packet sent by the mobile node, depending on its location. In the next section, we will use these parameters to, finally, compute the node’s position.

### 3.5. Position Computation

Now that we have the path loss model for the three best anchor nodes, we can convert the RSSI value of the packet sent by the mobile node and received by the anchors through an adaptation of Equation (2):

(11) $$\begin{array}{c} d_{ma}=10^{\left(\frac{R_{0}-R_{ma}}{10\times \eta_{ma}}\right)};\;\;\;\;\;\;\;\;d_{mb}=10^{\left(\frac{R_{0}-R_{mb}}{10\times \eta_{mb}}\right)};\;\;\;\;\;\;\;\;d_{mc}=10^{\left(\frac{R_{0}-R_{mc}}{10\times \eta_{mc}}\right)} \end{array}$$

where $d_{ma}$ is the estimated distance between the mobile node and the Anchor${}_{a}$, and so on.

Since the real positions of the anchors are known in advance and the distances are estimated using the previous equations, we can finally compute the mobile node position. For this, each anchor will have a circle of radius equal to the estimated distance, and the final target position will correspond to the intersection of the three circles. However, since we will have inaccuracies in our distance estimations, due to the RSSI variation [9], the formed circles often do not have a single intersection. To minimize this problem, we use the least-squares method [9,37] to optimize the position computation, as follow:

(12) $$f_{i}\left(x,y\right)=d_{mi}-\sqrt{{\left(x_{m}-x_{i}\right)}^{2}+{\left(y_{m}-y_{i}\right)}^{2}}$$

(13) $$min\left(x,y\right)=min\sum_{i=1}^{m}{\left[f_{i}\left(x,y\right)\right]}^{2},\;\;\;\;m\geq 3$$

However, it is difficult to resolve directly. To simplify, we can define the Equation (12) as $fi(x_{m},y_{m})=0$ and squared both sides to obtain:

(14) $${x_{m}}^{2}+{y_{m}}^{2}-2x_{m}x_{i}-2y_{m}y_{i}={d_{mi}}^{2}-{x_{i}}^{2}-{y_{i}}^{2}$$

Given the three anchors (Anchor${}_{a}$, Anchor${}_{b}$, Anchor${}_{c}$), their coordinates $\left(x_{a},y_{a}\right)$, $\left(x_{b},y_{b}\right)$, and $\left(x_{c},y_{c}\right)$, and the coordinate of mobile device $\left(x_{m},y_{m}\right)$. Then, if we set $w={x_{m}}^{2}+{y_{m}}^{2}$, we can organize as follows:

(15) $$\left\{\begin{array}{c} w-2x_{m}x_{a}-2y_{m}y_{a}={d_{ma}}^{2}-{x_{a}}^{2}-{y_{a}}^{2}\\ w-2x_{m}x_{b}-2y_{m}y_{b}={d_{mb}}^{2}-{x_{b}}^{2}-{y_{b}}^{2}\\ w-2x_{m}x_{c}-2y_{m}y_{c}={d_{mc}}^{2}-{x_{c}}^{2}-{y_{c}}^{2} \end{array}\right.$$

Then we can obtain an equation of the form AX=b:

(16) $$\begin{array}{c} A=\left[\begin{array}{ccc} 1 & -2x_{a} & -2y_{a}\\ 1 & -2x_{b} & -2y_{b}\\ 1 & -2x_{c} & -2y_{c} \end{array}\right] \end{array};\;\;\;\;\;\begin{array}{c} X=\left[\begin{array}{c} w\\ x_{m}\\ y_{m} \end{array}\right] \end{array};\;\;\;\;\;\begin{array}{c} b=\left[\begin{array}{c} {d_{ma}}^{2}{-x_{a}}^{2}-{y_{a}}^{2}\\ {d_{mb}}^{2}{-x_{b}}^{2}-{y_{b}}^{2}\\ {d_{mc}}^{2}{-x_{c}}^{2}-{y_{c}}^{2} \end{array}\right] \end{array}$$

Finally, the equation can be solved as a least-squares problem:

(17) $$X=({A^{T}A)}^{-1}A^{T}b$$

The result of the least-squares equation is the mobile node estimated position.

## 4. Performance Evaluation

In this section, we evaluate the performance of our proposed PoDME solution compared to the traditional fixed, model-based approaches found in the literature. We also evaluate other aspects of our solution such as the variation of the path loss exponent in a real-world scenario and the behavior of the data used for choosing the best anchors.

### 4.1. Testbed and Methodology

The main goal of our proposed IPS is to provide location information for small, battery-powered devices to be worn by people inside buildings, such as the elderly in retirement homes. These mobile devices are required to be operated by a single, small battery while also having other sensors. Thus, in our IPS, we decided to use the Bluetooth Low Energy (BLE) technology.

**Figure 4:**
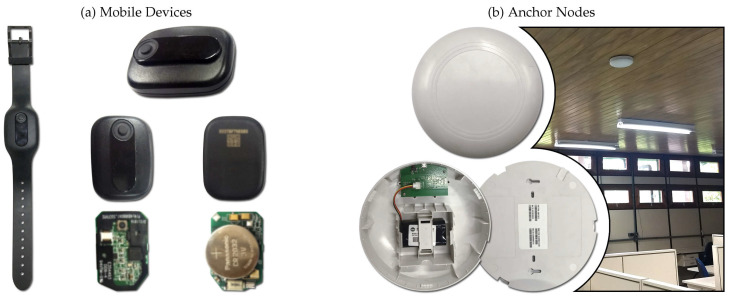
Testbed hardware: (**a**) mobile devices with Bluetooth communication; and (**b**) anchor nodes with Bluetooth and 900 Mhz communication (front, opened, back, and installed on the ceiling).

Furthermore, one of the premises of the hardware architecture was not to rely on the WiFi infrastructure of the building. Thus, to be able to send all of the gathered RSSI data to a central monitoring server, we developed a Bluetooth-based anchor. The testbed architecture works by mobile devices sending Bluetooth advertising packets every second and several anchors receive these packets. In our experiments, the longest communication distance between Bluetooth devices was 25 m. These anchor nodes compute the RSSI of the received packets by Bluetooth and send them to a central device using long-range, 900 Mhz communication. The central device is connected to a server, which will locate the mobile nodes. Figure 4 shows our developed hardware that was used in the testbed.

**Figure 5:**
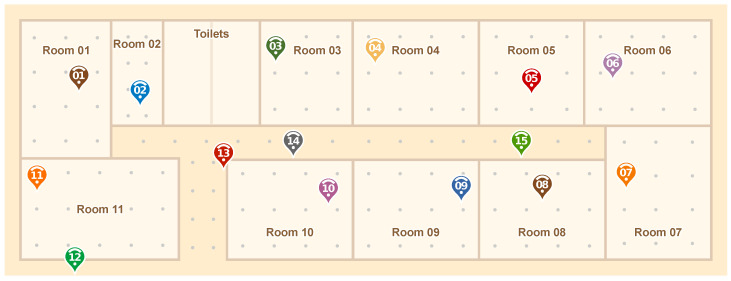
Testbed map: 11 rooms, 3 halls, and 15 anchor nodes. 100 packet samples collected from 150 test points.

To evaluate the performance of our proposed IPS solution, we carried out a large-scale experiment in a 43 × 15 m² area composed of 15 spaces (11 rooms and 3 halls), as shown in Figure 5. To cover the whole area, we deployed 15 anchor nodes fixed on the ceiling of the rooms in locations where it was somewhat convenient to connect them to the mains supply. It is important to note that to perform our Model Data Gathering, explained in Section 3.1, we used 8 different mobile devices and collected samples at 15 different locations. This was done to estimate the path loss exponents among anchors with a variety of signals from different devices.

However, to understand and evaluate our system results for the whole scenario, we gathered 100 packet samples from evenly spaced, 2 m apart locations, to a total of 150 different test points, which are the gray dots in Figure 5. For this, we used another three mobile devices that were different from the ones used in the Model Data Gathering. Thus, our testing was done with samples from a set of mobile devices that were not part of the Model Data Gathering. This step is important to ensure that the proposed system would work on new, never seen, mobile devices. Finally, it is important to note that these 150 test points data are not required for our PoDME solution, and were only used for performance evaluation purposes.

### 4.2. Signal Strength Analysis

**Figure 6:**
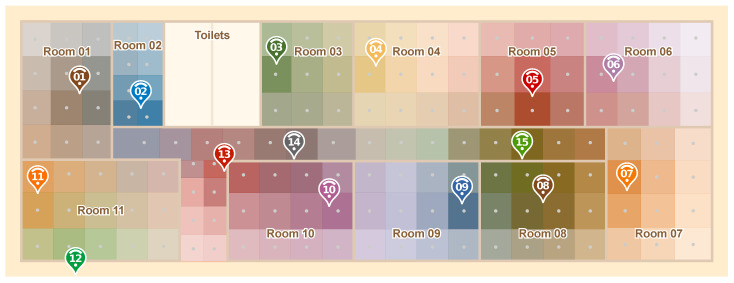
Signal characterization of the scenario based on the measurements made empirically.

To better understand the signal propagation behavior in our testbed environment, Figure 6 shows, for each test point, which anchor nodes received the packets from that point and at which signal strengths. For this, each anchor was given a different color. The stronger the color, the higher the signal strength. This map shows which points are covered by which anchors, and also gives us some insights into the behavior of the IPS. As we will see in the next sections, the lightest points did result in higher errors.

**Figure 7:**
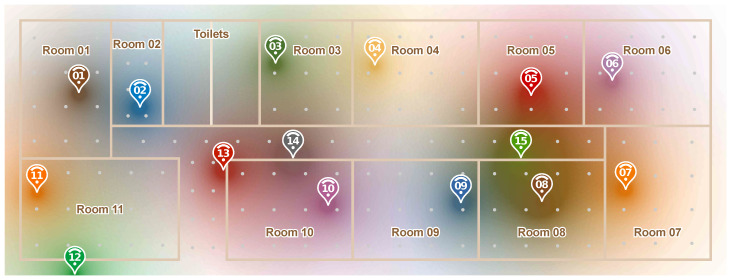
Signal characterization of the environment using the signal propagation model.

We then used these RSSI data to estimate the path loss exponent parameter of a fixed, log-distance propagation model. Our main goal is to allow the visualization of how a propagation model would compare to our real-world data. Thus, we implemented a simple signal propagation simulator. Figure 7 show the result of our simulation. It is interesting to see that, comparing both maps, we can notice that, in most parts, the colors seem to match. Even though this result is not scientific, it will help us to understand some of our obtained results in the next sections.

### 4.3. Path Loss Exponent Analysis

**Figure 8:**
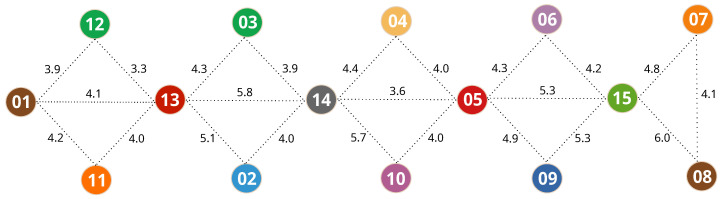
Path loss exponents among anchors computed from a real-world experiment. The lines are just a small subset of the connectivity among anchors and does not represent the full connectivity.

One of the key points of our PoDME solution is that the path loss model parameters change throughout the environment and, thus, using a fixed model would result in higher positioning errors. To depict these changes, Figure 8 shows some of the path loss exponents among the anchor nodes, as explained in Section 3.2, and based on the anchors of the map in Figure 5. It is important to note that the lines in this figure are just a small subset of the connectivity among anchors and does not represent the full connectivity.

As we can see, these values change considerably even among the neighbors of the same anchor. Even though this is a small set of the path loss exponents, we can see some cases in which the scenario affects the model parameters. For instance, focusing on Anchor${}_{3}$, we can see that its path loss exponent to Anchor${}_{14}$ is 3.9, while the exponent between Anchor${}_{3}$ and Anchor${}_{13}$ is higher, at 4.3. Both Anchor${}_{14}$ and Anchor${}_{13}$ are at similar distances from Anchor${}_{3}$, the main difference being that Anchor${}_{13}$ is shadowed by a corner and also by an extra wall, resulting in a higher path loss exponent.

### 4.4. Choosing the Best Anchors Parameters

Our main parameter for choosing the best anchor nodes is the RSSI values, as explained in Section 3.3. anchors with higher RSSI values are closer to the mobile device, which leads to lower distance estimation errors due to the model inaccuracies. To better understand the impact of the choice based on this criterion, for each sample to be located in our experiment, we computed the device’s position using all possible combinations of 3 anchors. For each computed position, we saved the positioning error and the average RSSI value among the three used anchors.

**Figure 9:**
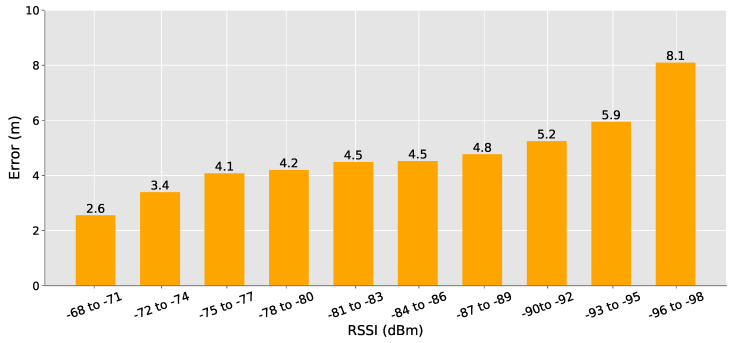
Positioning error by average RSSI. The farther the anchor nodes, the higher the positioning error. Even slightly farther anchor nodes can increase the positioning error by almost 1 m.

Figure 9 shows our results from the experiment. As expected, anchors with the highest RSSI values have the lowest average positioning error. However, one interesting aspect that we noticed after analyzing the graph, is that the difference between the first and the second bars are higher than expected. It means that getting slightly farther anchors can increase the positioning error by almost 1 m. It does show that our priority should be using the closest anchors.

Another key aspect of Choosing the Best Anchors is the equilateral triangle similarity checker, the ∆ shown in Equation (7). As mentioned, we noticed that even for closer anchors, when their positions were somewhat collinear, it resulted in considerably higher errors. Again, to better understand the impact of the anchors choice based on this criterion, for each sample to be located in our experiment, we computed the device’s position using all possible combinations of 3 anchors. For each computed position, we saved the positioning error and our equilateral triangle similarity (∆).

**Figure 10:**
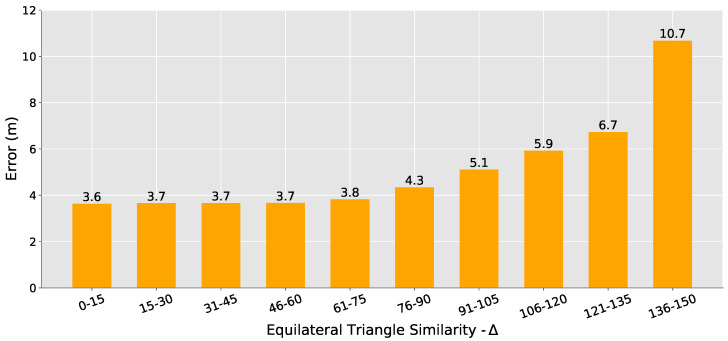
Positioning error by equilateral triangle similarity. The closer ∆ is from zero, the closer the anchor nodes are to form an equilateral triangle. For ∆ between 0 and 75, the error does not change significantly.

As shown in Figure 10, we can see that for ∆ between 0 and 75, we have an average error that does not change significantly, always below 4 m. However, after this value, the greater the ∆, the greater the average error. After analyzing these experiments and conduct some tests, we observed that combining these two criteria (RSSI and ∆) would not yield the best results, since they have different behaviors. Thus, we decided to use the ∆ value as a filter and established a threshold of 75 for allowing a set of three anchors to be used in the positioning while prioritizing the closest anchors.

### 4.5. Positioning Error Analysis

As mentioned, for our experiment, we captured RSSI samples in all of the points of the scenario to allow a fair comparison of the evaluated methods. For all measurements, we saved their correct positions on the map where the measurement was made, thus allowing us to compare the position estimated by the models and the actual point position.

We compared our PoDME solution to three variations of a fixed model-based IPS. These variations use the log-distance path loss propagation model, the same used in our solution, but instead of using a dynamic path loss exponent (η), they use a fixed value for all scenarios. It is important to note that the exponent value was computed based on the collected RSSI samples and we confirmed that it was the best possible fixed exponent, i.e., the one that resulted in the smallest errors. Finally, the main difference between these three variations and our solution is the choice of the anchors used for the position computation. While in our solution we used our method explained in Section 3.3, in the fixed model variations we used other possible solutions found in the literature:

1. Using 3 anchors with the highest RSSI values
2. Using 4 anchors with the highest RSSI values
3. Using all anchors

Using 3 [3,12,33] or all anchor nodes [15,38,39] in the position computation is a solution commonly found in the literature. However, the main reason we also experimented using the 4 anchors with the highest RSSIs, is that it could be a simple solution for the problem of anchors with collinear positions. Thus, as we will see, it resulted in considerably better performance when compared to using 3 anchors.

**Figure 11:**
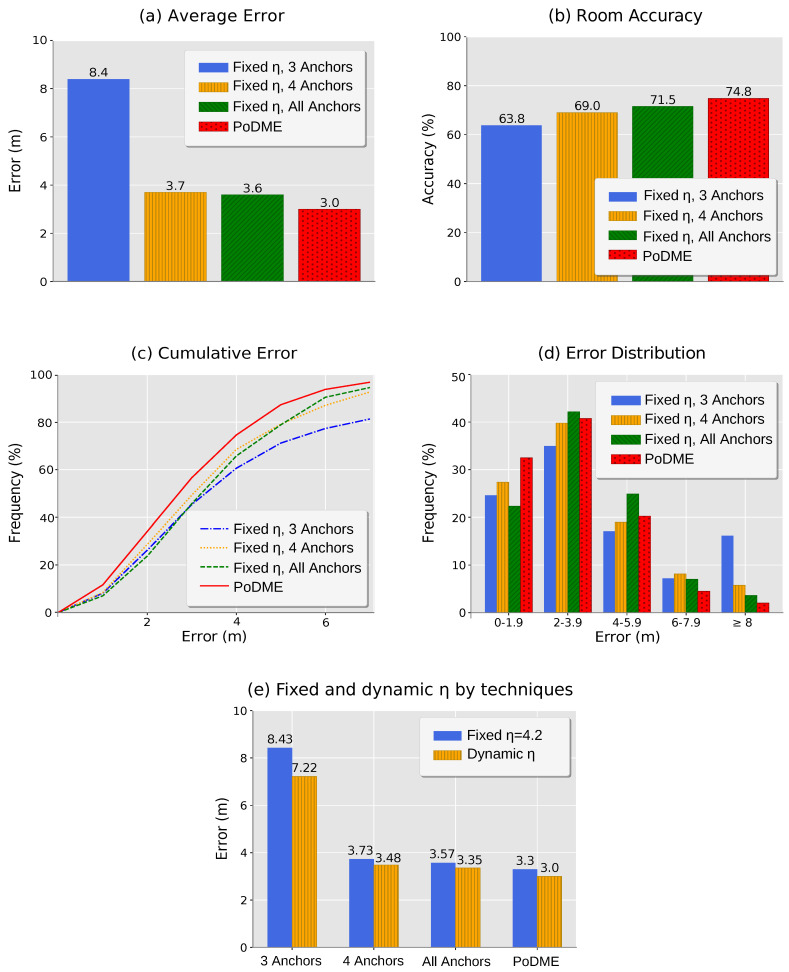
Positioning error analysis.

We first evaluate the average positioning error for each of the methods. As shown in Figure 11a, PoDME resulted in an average error of 3 m, being the smallest error when compared to the other approaches. As mentioned, we used a fixed path loss exponent for the fixed model variations. In these cases, the value was η = 4.2, obtained through the gathered data. Our solution used the dynamic path loss exponents values chosen based on the region of the three anchors with the highest RSSI values.

The worst results were obtained by the fixed, model-based method using information from 3 anchors with the highest RSSI values. As mentioned, in some cases, the chosen anchors are located in such a way that make their positions somewhat collinear, increasing considerably the error of the position estimations. As we can also see in Figure 11a, when we consider just one more anchor in the position computation we have a reduction of more than half of the error.

Figure 11b shows the average room accuracy. The room accuracy evaluation was done comparing whether the position estimated by each method was within the room limits where the measurement was performed. Since all of the models do not take into consideration the walls of the scenario, it is common for test points near walls to be located outside their rooms. Furthermore, small regions such as halls, also impact this metric, since the positioning error is greater than their width. However, as shown in Figure 11b, our proposal resulted in a greater room accuracy of 74.8%. As we will discuss in our conclusions, in our future work we intend to use a model that also takes into consideration the walls of the area, aiming at improving this metric.

The results obtained by our solution show that contrary to what it may seem initially if we continue using only 3 anchors, but considering better criteria for choosing these anchors, we can considerably reduce the total average error of the system. In Figure 11c, the curve of our solution (red line) grew the fastest. This means that our results contain the highest number of mobile devices with the smallest positioning error when compared to the other approaches. In Figure 11d, we can see that our approach contains most of the positioning errors between 0–4 m, although part of the samples was still located with higher errors. This happens due to regions of the map that are covered by only 3 anchors, not allowing the choice of other sets that could help in reducing the positioning error.

To evaluate the impact of the value used as the path loss exponent, in Figure 11e, we can see the average positioning error in relation to the use of a fixed path loss exponent value or the use of our proposed dynamic value. These results show that our proposed dynamic model was able to improve the accuracy of all evaluated variations of anchor choice. We can also see that our proposed PoDME solution takes advantage of not only the dynamic model but also the anchors choice, i.e., both aspects are responsible for improving the accuracy of the solution.

To better understand the behavior of the errors throughout the evaluated scenario, Table 3 shows the average error obtained by each approach for all rooms in the environment. We can see that the smallest errors per room vary a lot according to the approach used, that is, each room has its smallest error with different approaches. When we use 3 anchors, most measurements in Room 04 have the anchor nodes 3, 4, and 5 with the strongest signal power values. In this case, their organization on the map results in a high positioning error, as can be seen in Table 3. When we add an additional anchor node, the positioning error is drastically reduced. In our solution, even using only three anchors we obtain a low error as we choose the best anchors that help in the positioning calculation.

In most cases, our PoDME solution resulted in positioning errors close enough to the smallest error between all approaches. We use values in bold to facilitate comparison. The rooms with higher errors were rooms 06 and 07, on the right side of the map. The main reason for this is the lack of anchors coverage in some areas of these rooms, as depicted previously in Figure 7.

**Figure 12:**
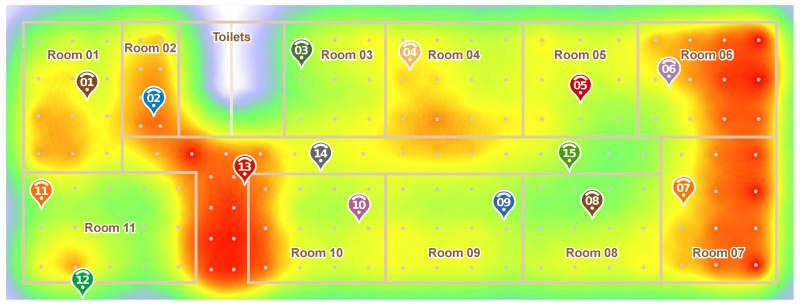
Heatmap of the average errors for each test point in the scenario.

Finally, in order to better visualize the data in Table 3, Figure 12 shows a heatmap of the errors in the whole scenario. Darker red colors indicate the areas with the highest errors. In this heatmap, we can notice another problematic region of the scenario, which is the hall near Anchor${}_{13}$. In this area, especially towards the bottom of the map, the other anchors (besides 13), are far from the hall which, combined with the walls in the area, resulted in a worse performance. This lack of anchors coverage can also be noticed in Figure 7.

## 5. Applicability of the Proposed Solution

It is known that fingerprint-based solutions [21,22,23,40] are among the most precise solutions for IPSs. However, they require an extensive training phase, making them basically impractical for medium to large-scale settings. One can argue that our proposed PoDME solution also has a training phase, which would be the Model Data Gathering (explained in Section 3.1). However, in our solution, we do not train all possible reference points of the environment but, rather, only a single point for each anchor.

As a comparison, in the experiments explained in Section 4 and depicted in Figure 5, we only needed to collect some samples from 15 different locations to generate our model. This can be done in fewer than 15 minutes since it does not require many samples. For a fingerprint-based solution, it would be required to train at least all of the 150 tested locations in our experiments. It took us several days to have unimpeded access to the rooms and collect enough data from all of the points. Also, the number of reference points increases drastically as the scenario increases.

In addition, our Model Data Gathering could be done automatically by the anchors, by modifying them to send data packets among themselves, as done by some works in the literature [31,32]. However, we do not think that this would be the best solution, since the signal strengths from packets exchanged between the anchors, which are all located in the ceiling, would differ significantly from the packets sent by the mobile nodes, which would be located in a mid-height. The main reason for this is the interference caused by the ceiling itself. Thus, we argue that our proposed Model Data Gathering would yield the best results, with little to no extra work when compared to other model-based IPSs.

Finally, as in most model-based IPSs [9,12,13,33], our solution also requires the prior knowledge of the anchor nodes positions, which is an extra step not required by fingerprint-based solutions. However, this information can be easily obtained through the site plan, since these positions do not need to be GPS-based. Also, if we aim at showing the location of the mobile nodes in a map application, this map would already be available.

## 6. Conclusions

In this paper, we propose and evaluate a new model-based IPS, in which the parameters of the model are dynamically estimated using the RSSI information from the best anchor nodes that received the packet sent by the mobile device we want to locate. Thus, for each packet sent by the mobile device, depending on its location, we have a different propagation model that will be used to estimate distances and, then, positions. The main goal of our proposed solution is to be implemented in medium to large-scale scenarios, in which a fingerprint-based solution would be hard or unfeasible to train and, also, a fixed, model-based solution would result in higher errors due to the fixed parameters of the model for the whole scenario. We also aim at applications in which mobile devices are highly energy-efficient such as small, battery-powered, sensor-based smartwatches, to be easily worn by people inside buildings such as the elderly in retirement homes.

Thus, we implemented a complete large-scale, Bluetooth-based testbed using custom-made hardware to evaluate the performance of our solution and compare it to traditional fixed, model-based solutions used by the current literature. Our experiments show a significant contribution in two of the main parts of our solution: the best anchors choice algorithm and the dynamic model estimation. When combined, our final solution resulted in an average error of 3 m, a 17% decrease when compared to the best experimented parameter of a fixed model-based IPS, that had an error of 3.6 m.

Even though we used the log-distance propagation model, our solution can also be applied to any other propagation model. In future work, we intend to experiment mainly with models that take into consideration the walls of the scenario. We also aim at proposing better algorithms for choosing the best anchor nodes by also taking advantage of the walls’ information. Finally, we will perform additional experiments to propose better solutions for the tested points located in small rooms, halls, and areas with less anchors coverage, since these were the areas that resulted in greater positioning errors and lower room accuracies. 

## Figures and Tables

**Table 1:** RSSI values between anchor nodes. Gathered by physically positioning a mobile node near an anchor node and sending packets that will be received by all other nearby anchors. This step is repeated for all anchor nodes in the scenario.

Anchor${}_{\mathrm{neighbor}}$	Anchor${}_{1}$	Anchor${}_{2}$	Anchor${}_{3}$	Anchor${}_{4}$	Anchor${}_{5}$
**Anchor** ${}_{1}$	-	−89	−90	−85	−77
**Anchor** ${}_{2}$	−89	-	−75	-	−80
**Anchor** ${}_{3}$	−90	−75	-	−73	-
**Anchor** ${}_{4}$	−85	-	−73	-	−82
**Anchor** ${}_{5}$	−77	−80	-	−82	-

**Table 2:** Path-loss exponents among anchor nodes calculated through the Equation (3) using the RSSI values from the Table 1.

Anchors${}_{\mathrm{neighbors}}$	Anchor${}_{1}$	Anchor${}_{2}$	Anchor${}_{3}$	Anchor${}_{4}$	Anchor${}_{5}$
**Anchor** ${}_{1}$	-	4.8	4.4	3.8	4.3
**Anchor** ${}_{2}$	4.8	-	4.2	-	5.1
**Anchor** ${}_{3}$	4.4	4.2	-	5.4	-
**Anchor** ${}_{4}$	3.8	-	5.4	-	5.9
**Anchor** ${}_{5}$	4.3	5.1	-	5.9	-

**Table 3:** Table with average error per room comparing the different approaches, highlighting the smallest mistakes compared to our approach.

Room	3 anchors		4 anchors		All anchors		PoDME
	Fixed η	Dynamic η	Fixed η	Dynamic η	Fixed η	Dynamic η	
Room 01	3.96	**3.10**	3.70	**3.10**	3.72	**3.10**	**3.12**
Room 02	3.30	**2.86**	3.90	3.07	3.90	3.07	**3.00**
Room 03	**2.54**	2.64	3.46	2.77	4.02	3.01	**2.64**
Room 04	48.52	35.21	6.27	5.50	**3.59**	3.98	**3.67**
Room 05	3.47	3.06	3.26	**2.97**	3.54	3.37	**3.00**
Room 06	3.58	4.31	**3.13**	4.13	4.29	4.13	**4.32**
Room 07	3.86	4.63	3.34	4.42	**2.75**	4.21	**4.29**
Room 08	2.77	3.16	**2.38**	2.49	3.07	2.61	**2.39**
Room 09	5.88	6.21	3.87	3.82	**2.60**	2.63	**2.60**
Room 10	4.51	3.35	3.90	**2.89**	3.23	**2.58**	**2.70**
Room 11	2.76	**2.37**	3.20	2.86	3.20	3.56	**2.24**
Hallway 1	7.24	8.47	**3.38**	3.45	4.49	3.92	**3.39**
Hallway 2	3.25	2.73	2.22	**2.02**	3.34	2.18	**2.23**
Hallway 3	5.5	7.43	6.02	4.37	5.69	**4.32**	**3.86**
Average	8.43 m	7.22 m	3.73 m	3.48 m	3.57 m	3.35 m	3.00 m
